# Genetic and Environmental Influences on Gambling: A Meta-Analysis of Twin Studies

**DOI:** 10.3389/fpsyg.2017.02121

**Published:** 2017-12-05

**Authors:** Yan-Hua Xuan, Shu Li, Rui Tao, Jie Chen, Li-Lin Rao, X. T. Wang, Rui Zheng

**Affiliations:** ^1^Key Laboratory of Behavioral Science, Institute of Psychology, Chinese Academy of Sciences, Beijing, China; ^2^Department of Psychology, University of Chinese Academy of Sciences, Beijing, China; ^3^Key Laboratory of Mental Health, Institute of Psychology, Chinese Academy of Sciences, Beijing, China; ^4^Department of Psychology, University of South Dakota, Vermillion, SD, United States

**Keywords:** gambling, heritability, meta-analysis, twin studies, disordered vs. general gambling

## Abstract

Disentangling the genetic and environmental influences of gambling is important for explaining the roots of individual differences in gambling behavior and providing guidance for precaution and intervention, but we are unaware of any comprehensive and systematic quantitative meta-analysis. We systematically identified 18 twin studies on gambling in the meta-analysis. The correlation coefficients within monozygotic (MZ) and dizygotic (DZ) twins, along with the corresponding sample size, were used to calculate the proportion of the total variance accounted for by additive genes (A), dominant genes (D), the shared environment (C), and the non-shared environment plus measurement error (E). We further assessed the moderating effects of gambling assessment (symptom oriented assessment vs. behavior oriented assessment), age, and sex. The whole sample analyses showed moderate additive genetic (a^2^ = 0.50) and non-shared environmental influences (e^2^ = 0.50) on gambling. The magnitude of the genetic influence (a^2^) was higher for disordered gambling assessed with symptom oriented assessment (53%) than for general gambling assessed with behavior oriented assessment (41%). Additionally, the magnitude of the genetic influence (a^2^) was higher for adults (53%) than adolescents (42%). Genetic influence (a^2^) was greater for male (47%) gambling than female (28%) gambling. Shared environment had noticeable effects on female gambling (c^2^ = 14%) but zero effect on male gambling. In conclusion, gambling behavior was moderately heritable and moderately influenced by non-shared environmental factors. Gambling assessment, age, and sex significantly moderated the magnitude of genetic and environmental influences on gambling. Note that the number of studies might serve as a limitation.

## Introduction

Game-based gambling dates far back in human history as an almost universal activity, in that about 70–90% of people gamble at some time in their lives (Shaffer et al., 1999; Welte et al., 2014). While most people gamble with few or no negative consequences, a small yet significant proportion of people (around 1–2%) develop disordered gambling behaviors that result in harm to themselves, their social network, or society (Walker and Dickerson, 1996; Abbott and Clarke, 2007). According to the fifth edition of the Diagnostic and Statistical Manual of Mental Disorders (DSM-V; American Psychiatric Association, 2013), disordered gambling is an addictive mental disorder classified in the “Addiction and Related Disorders” category. Prior research has attempted to determine the relative roles of genetic and environmental factors in gambling (Eisen et al., 1998; Winters and Rich, 1998; Potenza et al., 2005; Anokhin et al., 2009; Slutske et al., 2009, 2011, 2013; Beaver et al., 2010; Giddens et al., 2011; Blanco et al., 2012; Tuvblad et al., 2013; Richmond-Rakerd et al., 2014; Slutske and Richmond-Rakerd, 2014; Vitaro et al., 2014; Xian et al., 2014) and some reviews qualitatively summarized the related twin studies of gambling (Shah et al., 2005; Gyollai et al., 2013); however, we are unaware of any comprehensive and systemic quantitative meta-analysis.

Twin studies have examined the genetic and environmental influences of gambling, but the published heritability estimates have varied dramatically between different studies (from about 70 to 0%). A small proportion of studies indicated that gambling is primarily influenced by genetic factors. For example, Beaver et al. (2010) found that genetic factors explained approximately 70% of the variance in gambling. Other studies indicated a moderate genetic influence on gambling. Eisen et al. (1998) concluded that inherited factors explained between 35 and 53% of reported pathological gambling. Likewise, Slutske et al. (2011) concluded that genetic factors explained 49.2% of disordered gambling, as defined by DSM-IV, and 54.4% of disordered gambling, as defined by SOGS (South Oaks Gambling Screen). Some other studies indicated that gambling is influenced totally by environmental factors. For example, Slutske and Richmond-Rakerd (2014) found that gambling was influenced by both non-shared and shared environmental factors and that genetic factors played a negligible role in the occurrence of gambling.

There may be several explanations for the heterogeneity of the results from existing twin studies about gambling. First, two types of gambling assessments are available: symptom oriented assessment for disordered gambling (problem gambling) and behavior oriented assessment for non-addictive gambling (non-problem gambling) (Petry, 2000; Joukhador et al., 2004; Abbott and McKenna, 2005). Disordered gambling/problem gambling is an addictive mental disorder classified in the group termed “Addiction and Related Disorders” in DSM-V (Petry et al., 2005; Kessler et al., 2008; O’Brien, 2011). A previous review on addictions, such as drug abuse and alcoholism, indicated a moderate to high genetic influence (30–70%) (Agrawal and Lynskey, 2008) Thus, we assumed that disordered gambling assessed with symptom oriented assessment might be more largely influenced by genetic factors than general gambling assessed with behavior oriented assessment. Second, the heritability of gambling may change with age. Previous research has indicated that the effects of shared environments, such as family, tended to have a decreasing influence throughout development along with a concomitant increase in the influence of heritability and non-shared environmental effects on some behaviors (phenotypes) (Plomin, 1986; Loehlin, 1992; Miles and Carey, 1997; Bergen et al., 2007), but the results have not been consistent between different research fields (McCartney et al., 1990; Bezdjian et al., 2011). So, examining whether the influence of genetic and environmental factors in gambling varies at different time points throughout development, that is, at different ages, is necessary. Third, whether the heritability of gambling differs between the sexes remains unknown. Previous studies found that men were more likely to engage in gambling, seemingly because they took more risks and had lower levels of impulsive coping than women (Wong et al., 2013; Welte et al., 2014). Given this sex difference on a phenotypic level, it is also important to examine whether the magnitude of genetic and environmental effects differs between males and females.

These previous contradictory findings suggest the necessity of conducting reviews of twin studies in order to provide a clearer and more comprehensive picture of the magnitude of genetic and environmental influences on gambling. A few literature reviews of gambling have summarized the existing twin studies on gambling. These previous reviews were informative and summarized the related twin studies as suggesting a genetic influence on gambling (Raylu and Oei, 2002; Shah et al., 2005). However, these reviews did not provide information about the magnitude of the genetic and environmental influences. Walters (2001) conducted a meta-analysis to estimate the genetic and environmental influences of gambling and found a small but significant difference between the correlation indices of MZ and DZ twins, indicating the influence of heritable factors. However, his study only included two twin studies (further analysis was confined to family studies), making it difficult to reach a comprehensive conclusion.

Considering the heterogeneity of the results of previous twin studies and the shortage of existing reviews on gambling, synthesizing the existing studies to get a clear conclusion about the relative magnitude of the genetic and environmental influences is necessary. Therefore, we conducted this meta-analysis to clarify the influences of heritable genes, shared environmental factors, and unique environmental factors on gambling. Overall, the purpose of the present review was: (1) to synthesize and consolidate the existing twin literature on gambling to provide a clear and comprehensive conclusion; (2) to test the possible moderators, including gambling assessment, age, sex, and to clarify their influence on the genetic and environmental effects on gambling.

## Methods

### Background Information about Twin Design

The studies we examined in this meta-analysis were twin studies which compared the correlation indices of identical (monozygotic; MZ) and non-identical (dizygotic; DZ) twins on gambling. By using the classic twin experimental design, trait variance can be partitioned into its genetic and environmental components. MZ twins who share all their genes are compared with DZ twins who, on average, share half of their genes (Plomin, 1986). The magnitude of the additive genetic influences (A) and that of the dominant (non-additive) genetic influences (D) constitute the amount of variance between individuals that is due to genetic differences. If the genetic influences are additive, the effects of the alleles from different loci are independent and add up to influence the likelihood of developing a specific trait. If genetic influences are non-additive, the alleles interact with one another to influence the likelihood of developing that trait. In addition, the environmental influence consists of two components: shared environment and non-shared (unique) environment. Shared environmental variance (C) results from environmental influences shared within twin pairs, such as the family environment, parental style, and socio-economic status. Non-shared environmental variance (E) is due to environmental factors unique to each twin, such as idiosyncratic experiences and unshared peers and also includes measurement error. Note that it is not possible to examine the effects of “C” and “D” simultaneously, because their effects are confounded when examined together (Martin et al., 1978). If MZ twins are found to be more similar than DZ twins on a particular trait, the effect of genes would be indicated.

### Data Collection-Search Strategy, Inclusion Criteria, and Exclusion Criteria

We searched for twin studies on gambling in the following databases before April 1, 2017: PubMed, PsycINFO, Science Direct, Web of Science, and EBSCO. The search terms used included “twin” crossed with “gamble,” or “twin” crossed with “gambling.” The reference section of each empirical and review article was closely examined to identify any study that might have been missed or was published before these databases were established. Information about relevant published and unpublished data, such as unpublished doctoral dissertations and conference papers, was also collected and reviewed by contacting researchers through e-mail. This strategy yielded a total of 77 studies, of which 8 were reviews or meta-analyses.

These studies were examined to check their relevance according to the inclusion criteria mentioned below: first, only studies specifically examining gambling were included. Second, only studies that used twin samples and compared the correlation index in MZ twins and DZ twins to investigate the genetics of gambling were included. The effect sizes used in this meta-analysis were intraclass correlations or tetrachoric correlations for the MZ and DZ twins that were reported in the studies (Burt, 2009). If the study reported results through continuous measures and dichotomous measures (i.e., diagnostic symptom counts versus diagnoses), we chose the continuous index because considerable power and information are lost when sub- and suprathreshold variations in diagnostic status are collapsed into a dichotomous diagnostic variable (Krueger and Finger, 2001). These effects sizes were analyzed in model-fitting programs that estimate the relative contribution of genetic and environmental influences.

Fifty-nine studies were excluded from the meta-analysis for the following reasons: first, we excluded studies that did not provide the sample size (n) or the correlation indices for MZ and DZ twins. Another reason for exclusion was non-independent samples. Sample effect sizes were considered non-independent for several reasons. Several authors examined more than one dependent measure of the phenotype in the same sample either within a publication (e.g., Slutske et al., 2011) or across multiple publications (e.g., Richmond-Rakerd et al., 2014). Experts on meta-analysis have several suggestions for dealing with non-independent samples, including averaging the effect sizes of different dependent measures, selecting one measure (presumably the best measure) using the largest sample and omitting the others, or averaging the effect sizes when the samples in question are identical in size, and otherwise choosing the effect size from the largest sample (Lipsey and Wilson, 2001; Rhee and Waldman, 2002). Sullivan et al. (2003) recommended including only the most recent studies into the meta-analysis, if multiple publications used the same samples. We made use of the following strategy: if the sample sizes were similar, we used weighted averages to compute the study effect size (i.e., the sample size was used to weigh the contribution of a given effect size to average the effect size). If the sample sizes were not similar and the non-independent samples did not vary by age or method, the largest sample was chosen.

In the end, after accounting for non-independence of the samples, we identified 18 twin studies on gambling. These twin studies included in the meta-analysis are listed separately by sample, along with their effect sizes, measurement, assessment method, sample age, sample sex, and number of pairs by zygosity in the Supplementary Materials.

### Data Analysis and Model Fitting

Structural equation modeling was used to perform the genetic model-fitting analyses in Mx (Neale et al., 2003). Mx uses maximum-likelihood model-fitting techniques to fit models to the observed correlation matrices. We compared the ACE, AE, CE, and ADE models for all the data. The fit of each model, as well as of competing models, was assessed through chi-square difference tests and Akaike’s information criterion (AIC). Among the competing models, the one with the lowest AIC and the lowest chi-square value relative to its degrees of freedom was considered to be the best fitting model.

### Assessment of Potential Moderators

We examined whether gambling assessment (i.e., symptom oriented assessment vs. behavior oriented assessment), age (i.e., adolescents vs. adults), or sex (i.e., male vs. female) were significant moderators by contrasting the fit of a model in which the parameter estimates were constrained to be equal across the levels of the moderators with the fit of the model in which the parameter estimates were free to vary across levels of moderators. If the fits of the two models were significantly different (differences determined by chi-squares), this indicated that the moderation effect on the genetic and environmental parameter estimates was significant. It should be noted that non-significant results may have resulted from a lack of power and little variability in the levels of the moderator.

#### Gambling Assessment

Gambling can be assessed through symptom oriented assessment (disordered gambling) or through behavior oriented assessment (general gambling). Because all the studies used in this meta-analysis were performed on general populations, rather than treatment-seeking populations, symptom oriented assessment was used by the original authors of the relevant studies to identify symptoms of disordered gambling, which is defined as “persistent and recurrent maladaptive gambling behavior that disrupts personal, family, or vocational pursuits” (American Psychiatric Association, 1994). The symptom oriented assessment for disordered gambling include the Diagnostic and Statistical Manual (DSM–III–R, DSM–IV, and DSM-V), the South Oaks Gambling Screen (SOGS; Lesieur and Blume, 1987), the Gambler’s Anonymous Scale (GA20; Ursua and Uribelarrea, 1998) and the Canadian Problem Gambling Severity Index (PGSI; Ferris and Wynne, 2001). In contrast, the behavior oriented assessment is not defined by a diagnosis but can be described as risk propensity and participation in gambling activities. Therefore, the studies that investigated general gambling typically assessed the gambling behavior by self-reports about involvement in popular gambling activities (e.g., lottery, electronic gaming machines, and card games) and laboratory tasks which measured the performance of individuals in simulated gambling situations, including the Iowa Gambling Task and the Balloon Analogue Risk Task. Because addiction is often largely influenced by genetic factors (Agrawal and Lynskey, 2008; Li and Burmeister, 2009; Agrawal et al., 2012), we further explored the potential moderation effect of gambling assessment to examine the difference in heritability.

#### Age

Because access to the raw data for each study was not possible, in our meta-analysis age was simplified into a categorical variable (i.e., adolescents and adults). Many countries define 18 as the legal age of adults, so we separated the participants into adults and adolescents based on this standard.

#### Sex

Because a sex difference in prevalence is known to exist (Wong et al., 2013; Welte et al., 2014), it is important to consider whether the magnitude of the genetic and environmental influences differs in males and females. Therefore, the present meta-analysis examined whether sex is a significant moderator of the results of behavioral genetics studies of gambling. We removed the results of opposite-sex twin pairs to assess the moderating effect of sex by comparing the correlation indices of the MZ and the DZ twins for males and females in the meta-analysis.

## Results

### Analysis of All Data

In the end, the analyses included 18 papers that met the inclusion criteria. Stem and leaf plot of the effect sizes from the twin studies is shown in Supplementary Materials. All of the twin studies were conducted in the general population rather than in treatment-seeking samples. The fits of the various models, as well as of competing models, were compared and the magnitudes of the genetic and environmental influences of the models were calculated using a structural equation model. The results are presented in **Table 1**.

**Table 1:** Standardized parameter estimates and fit statistics – inclusion of all data.

	Parameter estimate				Fit statistic			
Model	a^2^	c^2^	e^2^	d^2^	χ^2^	*df*	*p*	AIC
ADE	0.49	–	0.49	0.02	409.46	109	<0.001	191.46
ACE	0.50	0.00	0.50	–	409.57	109	<0.001	191.57
**AE**	**0.50**	**–**	**0.50**	**–**	**409.57**	**110**	**<0.001**	**189.57**
CE	–	0.38	0.62	–	756.11	110	<0.001	536.11

*Dashes indicate that data are not applicable. AIC = Akaike’s information criterion; A = addictive genetic effects; C = shared environmental effects; E = non-shared environmental effects; D = non-additive genetic effects. The best fitting model is in bold and underlined.*

The AE model was the best fitting model with the lowest AIC value and the lowest chi-square value relative to its degrees of freedom. Gambling behavior was moderately heritable (a^2^ = 0.50 [95% confidence interval (CI): 0.49–0.52]), and was also moderately influenced by non-shared environmental factors (e^2^ = 0.50 [95% CI: 0.48–0.51]).

### Assessment of Potential Moderators

**Table 2** shows the results of the analyses examining gambling assessment, sex, and age as moderators of the magnitude of the genetic and environmental influences of gambling. The chi-square difference between a model in which the parameter estimates were free to vary across the different levels of the moderators and a model in which the parameter estimates were constrained to be equal is presented in **Table 2** for each moderator.

**Table 2:** Standardized parameter estimates and fit statistics for the best fitting models in the moderation effect analysis.

Moderator variables	χ^2^	*df*	*p*-value	AIC	a^2^	c^2^	e^2^
**Gambling assessment**							
Parameters free	222.44	113	<0.001	-3.56			
Symptom oriented assessment					0.53	–	0.47
Behavior oriented assessment					0.41	–	0.59
Parameters constrained	266.22	115	<0.001	36.22	0.48	–	0.52
Difference in χ^2^	43.78	2	<0.001	39.78			
**Age**							
Parameters free	406.35	119	<0.001	168.35			
Adolescents					0.42	–	0.58
Adults					0.53	–	0.47
Parameters constrained	453.92	121	<0.001	211.92	0.49	–	0.51
Difference in χ^2^	47.56	2	<0.001	43.56			
**Sex**							
Parameters free	91.96	79	0.15	-66.04			
Male					0.47	–	0.53
Female					0.28	0.14	0.58
Parameters constrained	98.69	82	0.10	-65.31	0.45	0.00	0.55
Difference in χ^2^	6.74	3	<0.001	0.74			

*AIC = Akaike’s Information Criterion; A = addictive genetic effects; C = shared environmental effects; E = non-shared environmental effects; D = non-additive genetic effects.*

#### Gambling Assessment

**FIGURE 1:**
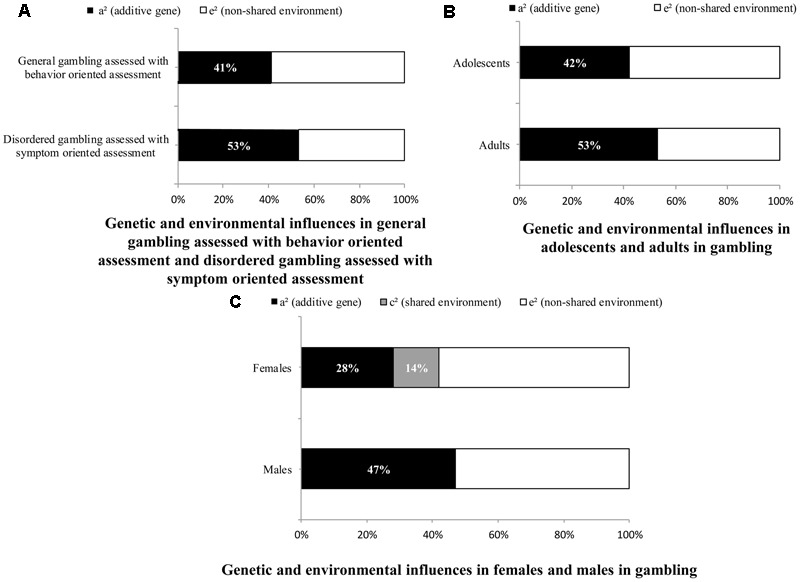
Genetic and environmental influences on gambling. **(A)** Indicates the proportions of the genetic and environmental influences on disordered gambling assessed with symptom oriented assessment and general gambling assessed with behavior oriented assessment; **(B)** indicates the relative magnitude of genetic and environmental influences on gambling for adolescents and adults; **(C)** indicates the relative contribution of genetic and environmental factors on gambling for females and males.

The meta-analysis included 11 papers that measured disordered gambling through symptom oriented assessment and 6 papers that measured general gambling through behavior oriented assessment. As shown in **Table 2**, the chi-square difference was significant for the gambling assessment [Δχ^2^(2) = 43.78, *p* < 0.01], indicating a significant difference in the magnitude of the genetic and environmental influences between disordered gambling assessed with symptom oriented assessment and general gambling assessed with behavior oriented assessment. The AE model was the best fitting model for both disordered gambling assessed with symptom oriented assessment (a^2^ = 0.53 [95% CI 0.50–0.55], e^2^ = 0.47 [95% CI 0.45–0.49]), and general gambling assessed with behavior oriented assessment (a^2^ = 0.41 [95% CI 0.38–0.44], e^2^ = 0.59 [95% CI 0.55–0.62]). When these two types of gambling were compared (**Figure 1A**), the magnitude of the genetic influence (a^2^) was higher for disordered gambling assessed with symptom oriented assessment. It should be noted that, in the symptom oriented assessment condition, we collapsed both self-reported and behavioral assessments of gambling, which might lead to potential problems.

#### Age

The meta-analysis included 14 papers on adults and 4 papers on adolescents. The chi-square difference test indicated that age was a significant moderator and the magnitudes of genetic and environmental influences on gambling in adolescents and adults were significantly different [Δχ^2^(2) = 47.56, *p* < 0.01]. The AE model was the best fitting model for adolescents (a^2^ = 0.42 [95% CI 0.39–0.44], e^2^ = 0.58 [95% CI 0.56–0.61]) as well as for adults (a^2^ = 0.53 [95% CI 0.51–0.55], e^2^ = 0.47 [95% CI 0.45–0.49]). The magnitude of the genetic influence (a^2^) was larger in adults than adolescents. A comparison of the effects of adolescents and adults on gambling is presented in **Figure 1B**. According to Hedges and Vevea (1998), using less than five effect sizes might result in random-effect tests that can only be regarded as approximate, caution should be taken when interpreting our result about the magnitude of the genetic influence in adolescents.

#### Sex

Because several studies examined only one sex and several studies did not report results separately by sex, the comparison of the results for males and females was performed after excluding these studies and finally included 13 papers. Chi-square difference tests of the differences were significantly different for males and females [Δχ^2^(3) = 6.74, *p* < 0.01]. The AE model was the best fitting model for males (a^2^ = 0.47 [95% CI: 0.43–0.52], e^2^ = 0.53 [95% CI: 0.48–0.57]), whereas the ACE model was the best fitting model for females (a^2^ = 0.28 [95% CI: 0.15–0.42], c^2^ = 0.14 [95% CI: 0.02–0.26], e^2^ = 0.58 [95% CI: 0.53–0.62]). **Figure 1C** showed that the magnitudes of genetic and environmental influences differed between males and females.

## Discussion

When all the available twin studies on gambling were analyzed together, the best fitting model was the AE model. On the basis of this analysis, there were moderate additive genetic (a^2^ = 0.50) and non-shared environmental influences (e^2^ = 0.50) on gambling. The influences of genetic and non-shared environmental effects (such as idiosyncratic experiences and unshared peers) were almost identical, and shared environment (such as the family environment) played a negligible role on gambling behavior in general.

With regard to the moderation effect, we found that gambling assessment, age, and sex significantly affected the genetic and environmental influences on gambling. The influence of genetic factors was higher for disordered gambling assessed with symptom oriented assessment (53%) than for general gambling assessed with behavior oriented assessment (41%). These results were in agreement with a previous qualitative review of twin studies, which also found a similar heritability of disordered gambling (50–60%) using the Vietnam Era Twin Registry (Lobo and Kennedy, 2009). Disordered gambling assessed with symptom oriented assessment was more influenced by genetic factors, which suggested that searching for specific genes associated with disordered gambling could be important for enabling precautions and providing corresponding interventions. Identifying the genotypes could be useful for early screening of at-risk individuals. In addition, because of the contribution of non-shared environment, the influence of an individual’s unique life experiences could be more important for general gambling than for disordered gambling.

Pathological gambling often co-occurs with pathological substance use (Langenbucher et al., 2001) and a high level of impulsivity (Bagby et al., 2007). As found in our meta-analyses, disordered gambling assessed with symptom oriented assessment was largely attributable to genetic factors. In light of the genetic basis of both substance use (McGue et al., 2000) and impulsivity (Kreek et al., 2005), future research could further explore the potential shared genetic predisposition underlying the covariance between gambling and these mental disorders.

Age was found to account for significant differences in the genetic and environmental influences on gambling; that is, the heritability of gambling behavior was lower in adolescents than in adults. The results were consistent with previous behavioral genetic research, which has generally found that heritability estimates of human behavioral traits increase with age (Plomin, 1986; Bouchard et al., 1996). Environmental influences explained more variance of gambling behavior in adolescents than in adults. That is, risk factors related to social environment (e.g., affiliation with peers) made larger contributions to the variance of occurrence and development of gambling in adolescents than in adults. The heritability was higher for adults, a finding which may be due to genetic predispositions which cause individuals to select environments that expose them to risk factors for the behavior (Plomin, 1986).

The meta-analysis found that sex was a significant moderator of the magnitude of genetic and environmental influences. The heritability of gambling was higher for men (47%) than for women (28%). Shared environment had noticeable effects on female gambling (c^2^ = 14%) but zero effect on male gambling. The results suggested that the variance in females’ gambling behavior was influenced by a greater magnitude of shared and non-shared environmental influences, which may be manipulated more easily than genetic factors when developing intervention strategies. Therefore, intervention programs targeting female gambling could benefit from focusing on external influences, such as family and peer groups.

There are several limitations to the current meta-analysis. First, the number of twin studies included in our meta-analysis might serve as a limitation. Due to the small literature suitable for the meta-analysis, it is difficult to draw a firm conclusion based on our data. Another limitation is that the results were based only on a low number of independent cohorts and all cohorts were from western countries. The countries from which the different samples were drawn have different policies toward gambling, which might influence the reported genetic and environmental estimates.

## Conclusion

Our meta-analysis aggregated the results of a number of previous twin studies and provided comprehensive estimates of the genetic and environmental influences on gambling. We confirmed that both additive genetic and non-shared environmental factors played important roles in gambling and identified the concrete scope of these genetic and environmental influences. We also found that the gambling assessment, age, and sex accounted for significant differences in the genetic and environmental influences on gambling. By identifying these moderators, our meta-analysis provided scientific evidence for early screening and further intervention for problem gambling.

## Author Contributions

Y-HX and RZ collected the study data, conducted the main statistical analysis, prepared and wrote the manuscript. SL, L-LR, and RT contributed to the writing of the manuscript and manuscript editing. JC contributed to the statistical editing. XW contributed to the manuscript editing.

## Conflict of Interest Statement

The authors declare that the research was conducted in the absence of any commercial or financial relationships that could be construed as a potential conflict of interest.
