# Supplementary material for: Genetic and Environmental Influences on Gambling: A Meta-Analysis of Twin Studies
Source: Front Psychol. 2017 Dec 5;8:2121. doi: 10.3389/fpsyg.2017.02121 (PMC5723410; doi:10.3389/fpsyg.2017.02121)
Supplement: Supplementary file 1 [file Table_1.DOCX]

**Supplementary Material**

Table S1. Overview of twin studies into gambling included in our meta-analysis

| Sample & study | Measurement | Assessment method | Age group | N | Relation-sex | Effect size | Inclusion |
| --- | --- | --- | --- | --- | --- | --- | --- |
| USA web-based general population sample | | | | | | | |
| (Blanco et al. 2012) | | | | | | | |
|  | Gambling frequency  (behavior oriented assessment) | Web-based questionnaire | 25.4 ±11.7 | 414 | MZ | 0.76 | Included- average |
|  |  |  |  | 159 | DZ | 0.57 | Included- average |
|  | DSM-IV criteria symptoms of disordered gamble  (symptom oriented assessment) |  |  | 414 | MZ | 0.83 | Included- average |
|  |  |  |  | 159 | DZ | 0.32 | Included- average |
| Vietnam Era Twin Registry | | | | | | | |
| (Eisen et al. 1998) | | | | | | | |
|  | DSM-III-R pathological gambling symptoms and disorder  (symptom oriented assessment) | Telephone interview | 33-53 | 1869 | MZ-M | 0.51 | Included- average |
|  |  |  |  | 1490 | DZ-M | 0.21 | Included- average |
| (Giddens et al. 2011) | | | | | | | |
|  | DSM-III-R pathological gambling symptoms and disorder  (symptom oriented assessment) | Telephone interview | 42±2.8 | 1874 | MZ-M | 0.62 | Included- average |
|  |  |  |  | 1498 | DZ-M | 0.40 | Included- average |
| (Potenza et al. 2005) | | | | | | | |
|  | DSM-III-R pathological gambling symptoms and disorder  (symptom oriented assessment) | Telephone interview | 42±2.8 | 1874 | MZ-M | 0.62 | Excluded (for repeated sample) |
|  |  |  |  | 1498 | DZ-M | 0.40 | Excluded |
| (Xian et al. 2014) | | | | | | | |
|  | Life-time DSM-III-R diagnoses  (symptom oriented assessment) | Interview | 42±2.8 | 1874 | MZ-M | 0.49 | Included- average |
|  |  |  |  | 1498 | DZ-M | 0.19 | Included- average |
| Australian Twin Registry Younger | | | | | | | |
| (Slutske et al. 2011) | | | | | | | |
|  | DSM-IV DG  (symptom oriented assessment) | Telephone interview | 32-43 | 347 | MZ-M | 0.49 | Included- average |
|  |  |  |  | 520 | MZ-F | 0.55 | Included- average |
|  |  |  |  | 227 | DZ-M | 0.21 | Included- average |
|  |  |  |  | 367 | DZ-F | 0.21 | Included- average |
|  |  |  |  | 414 | DZ-OS | 0.22 | Included- average |
|  | South Oaks gambling screen  (symptom oriented assessment) | Telephone interview |  | 347 | MZ-M | 0.64 | Included- average |
|  |  |  |  | 520 | MZ-F | 0.49 | Included- average |
|  |  |  |  | 227 | DZ-M | 0.19 | Included- average |
|  |  |  |  | 367 | DZ-F | 0.28 | Included- average |
|  |  |  |  | 414 | DZ-OS | 0.16 | Included- average |
| (Richmond-Rakerd et al. 2014) | | | | | | | |
|  | DSM-IV DG  (symptom oriented assessment) | Telephone interview | 32-43 | 347 | MZ-M | 0.49 | Excluded (for repeated sample) |
|  |  |  |  | 520 | MZ-F | 0.55 | Excluded |
|  |  |  |  | 227 | DZ-M | 0.21 | Excluded |
|  |  |  |  | 367 | DZ-F | 0.21 | Excluded |
|  |  |  |  | 414 | DZ-OS | 0.22 | Excluded |
| (Slutske et al. 2013) | | | | | | | |
|  | DSM-IV DG (categorical)  (symptom oriented assessment) | Structured telephone interview | 32–43 | 347 | MZ-M | 0.49 | Excluded |
|  |  |  |  | 520 | MZ-F | 0.53 | Excluded |
|  |  |  |  | 227 | DZ-M | 0.24 | Excluded |
|  |  |  |  | 367 | DZ-F | 0.22 | Excluded |
|  |  |  |  | 414 | DZ-OS | 0.23 | Excluded |
|  | DSM-IV DG (continuous)  (symptom oriented assessment) |  |  | 347 | MZ-M | 0.57 | Included- average |
|  |  |  |  | 520 | MZ-F | 0.57 | Included- average |
|  |  |  |  | 227 | DZ-M | 0.3 | Included- average |
|  |  |  |  | 367 | DZ-F | 0.28 | Included- average |
|  |  |  |  | 414 | DZ-OS | 0.15 | Included- average |
| (Slutske et al. 2009) | | | | | | | |
|  | Mean correlation for all 11 Gambling activities involvement  (behavior oriented assessment) | Structured telephone interview ; questionnaire | 37.7 ±0.04  (32–43) | 867 | MZ | 0.56 | Included-average |
|  |  |  |  | 1008 | DZ | 0.32 | Included- average |
|  | Versatility  (behavior oriented assessment) |  |  |  | MZ | 0.58 | Included- average |
|  |  |  |  |  | DZ | 0.28 | Included- average |
|  | Max frequency(days/year)  (behavior oriented assessment) |  |  |  | MZ | 0.48 | Included- average |
|  |  |  |  |  | DZ | 0.21 | Included- average |
|  | Max spent($/day)  (behavior oriented assessment) |  |  |  | MZ | 0.58 | Included-average |
|  |  |  |  |  | DZ | 0.31 | Included-average |
| (Slutske et al. 2010) | | | | | | | |
|  | DSM-IV DG  (symptom oriented assessment) | Telephone interview | 32-43 | 347 | MZ-M | 0.49 | Excluded (for repeated sample) |
|  |  |  |  | 520 | MZ-F | 0.55 | Excluded |
|  |  |  |  | 227 | DZ-M | 0.21 | Excluded |
|  |  |  |  | 367 | DZ-F | 0.21 | Excluded |
|  |  |  |  | 414 | DZ-OS | 0.22 | Excluded |
| National Longitudinal Study of Adolescent Health | | | | | | | |
| (Slutske and Richmond-Rakerd 2014) | | | | | | | |
|  | Any gambling involvement  (behavior oriented assessment) |  |  | 111 | MZ-M | 0.41 | Included –average |
|  |  |  |  | 130 | MZ-F | 0.50 | Included –average |
|  |  |  |  | 107 | DZ-M | 0.44 | Included –average |
|  |  |  |  | 93 | DZ-F | 0.59 | Included –average |
|  |  |  |  | 156 | DZ-OS | 0.38 | Included –average |
|  | Gambling activity count  (behavior oriented assessment) |  |  | 111 | MZ-M | 0.4 | Included –average |
|  |  |  |  | 130 | MZ-F | 0.47 | Included –average |
|  |  |  |  | 107 | DZ-M | 0.39 | Included –average |
|  |  |  |  | 93 | DZ-F | 0.49 | Included –average |
|  |  |  |  | 156 | DZ-OS | 0.46 | Included –average |
| (Beaver et al. 2010) | | | | | | | |
|  | 8 questions designed to measure serious gambling problems  (symptom oriented assessment) | Self-report | 11-20 | 324 | MZ | 0.685 | Included- average |
|  |  |  |  | 278 | DZ | 0.323 | Included- average |
| Los Angeles area | | | | | | | |
| (Tuvblad et al. 2013) | | | | | | | |
|  | Iowa Gambling Task  (behavior oriented assessment) | Laboratory experiment | 11-13 | 177 | MZ | 0.26 | Included |
|  |  |  |  | 166 | DZ | 0.14 | Included |
|  |  |  | 14-15 | 348 | MZ | 0.07 | Included |
|  |  |  |  | 473 | DZ | 0.12 | Included |
|  |  |  | 16-18 | 198 | MZ | 0.33 | Included |
|  |  |  |  | 251 | DZ | 0.16 | Included |
| United States | | | | | | | |
| (Winters and Rich 1998) | | | | | | | |
|  | High-action games (including lottery etc.) frequency  (behavior oriented assessment) | Questionnaire | 26.1 | 42 | MZ-M | 0.58 | Included |
|  |  |  |  | 33 | MZ-F | 0.50 | Included |
|  |  |  |  | 50 | DZ-M | 0.18 | Included |
|  |  |  |  | 30 | DZ-F | 0.43 | Included |
|  | Non-high-action games (including informal cards etc.) frequency  (behavior oriented assessment) | Questionnaire | 26.1 | 42 | MZ-M | 0.59 | Included |
|  |  |  |  | 33 | MZ-F | 0.33 | Included |
|  |  |  |  | 50 | DZ-M | 0.41 | Included |
|  |  |  |  | 30 | DZ-F | 0.23 | Included |
| American general population | | | | | | | |
| (Anokhin et al. 2009) | | | | | | | |
|  | BART  (behavior oriented assessment) | Laboratory experiment | 12.5±0.21 | 82 | MZ-M | 0.30 | Included |
|  |  |  |  | 87 | MZ-F | 0.18 | Included |
|  |  |  |  | 71 | DZ-M | -0.01 | Included |
|  |  |  |  | 49 | DZ-F | 0.06 | Included |
|  |  |  |  | 83 | DZ-OS | 0.24 | Included |
|  |  |  | 14.6±0.24 | 41 | MZ-M | 0.60 | Included |
|  |  |  |  | 57 | MZ-F | -0.11 | Included |
|  |  |  |  | 41 | DZ-M | 0.19 | Included |
|  |  |  |  | 24 | DZ-F | 0.22 | Included |
|  |  |  |  | 60 | DZ-OS | 0.08 | Included |
| Quebec Newborn Twin Registry | | | | | | | |
| (Vitaro et al. 2014) | | | | | | | |
|  | South Oaks Gambling Screen for adolescents (SOGS-RA) frequency of involvement in gambling  (symptom oriented assessment) | Self-rating questionnaire | 13 | 160 | MZ | 0.55 | Included |
|  |  |  |  | 119 | DZ | 0.18 | Included |
| Australian Twin Registry Cohort Ⅱ | | | | | | | |
| (Slutske et al. 2015) | | | | | | | |
|  | Gambling frequency for 11 different gambling activities in past year  (symptom oriented assessment) | Telephone interview | 37.7 | 319 | MZ-M | 0.50 | Included |
|  |  |  |  | 479 | MZ-F | 0.45 | Included |
|  |  |  |  | 211 | DZ-M | 0.27 | Included |
|  |  |  |  | 339 | DZ-F | 0.31 | Included |
|  |  |  |  | 376 | DZ-OS | 0.16 | Included |
|  | Disordered gambling  (symptom oriented assessment) | DSM-5 diagnosis | 37.7 | 319 | MZ-M | 0.42 | Included |
|  |  |  |  | 479 | MZ-F | 0.42 | Included |
|  |  |  |  | 211 | DZ-M | 0.23 | Included |
|  |  |  |  | 339 | DZ-F | 0.25 | Included |
|  |  |  |  | 376 | DZ-OS | 0.16 | Included |
| Beijing Twin Study (BeTwiSt) registry | | | | | | | |
| (Wang et al., 2016) | | | | | | | |
|  | Gambling subscale of Domain-Specific Risk-Taking Scale Across Seven Domains  (behavior oriented assessment) |  |  | 69 | MZ-M | 0.24 | Included |
|  |  |  |  | 82 | MZ-F | 0.15 | Included |
|  |  |  |  | 39 | DZ-M | 0.11 | Included |
|  |  |  |  | 50 | DZ-F | 0.31 | Included |

Table S2. Stem and leaf plot of the effect sizes (correlations) in twin studies

| Stem | Leaf | | | |
| --- | --- | --- | --- | --- |
|  | MZ twin pairs | | | DZ twin pairs |
|  | r=a²+c² | | r=0.5a²+c² | |
| Gambling | | | | |
| .8 | | 3 |  | |
| .7 | | 6 |  | |
| .6 | | 0249 |  | |
| .5 | | 0001556778889 | 79 | |
| .4 | | 0122578999 | 013469 | |
| .3 | | 033 | 011122289 | |
| .2 | | 46 | 11112233457888 | |
| .1 | | 58 | 1245666688999 | |
| .0 | | 7 | 68 | |
| -.0 | |  | 1 | |
| -.1 | | 1 |  | |
